# Radiation Sterilization of Anthracycline Antibiotics in Solid State

**DOI:** 10.1155/2013/258758

**Published:** 2013-11-03

**Authors:** A. Kaczmarek, J. Cielecka-Piontek, P. Garbacki, K. Lewandowska, W. Bednarski, B. Barszcz, P. Zalewski, W. Kycler, I. Oszczapowicz, A. Jelińska

**Affiliations:** ^1^Biofarm Sp. z o.o., Wałbrzyska 13, 60-198 Poznań, Poland; ^2^Department of Pharmaceutical Chemistry, Faculty of Pharmacy, Poznan University of Medical Sciences, Grunwaldzka 6, 60-780 Poznań, Poland; ^3^Institute of Molecular Physics, Polish Academy of Sciences, Smoluchowskiego 17, 60-179 Poznań, Poland; ^4^Department of Oncological Surgery II, Great Poland Cancer Centre, Garbary 15, 61-866 Poznań, Poland; ^5^Department of Modified Antibiotics, Institute of Biotechnology and Antibiotics, Starościńska 5, 02-516 Warsaw, Poland

## Abstract

The impact of ionizing radiation generated by a beam of electrons of 25–400 kGy on the stability of such analogs of anthracycline antibiotics as daunorubicin (DAU), doxorubicin (DOX), and epidoxorubicin (EPI) was studied. Based on EPR results, it was established that unstable free radicals decay exponentially with the half-time of 4 days in DAU and DOX and 7 days in EPI after irradiation. Radiation-induced structural changes were analyzed with the use of spectrophotometric methods (UV-Vis and IR) and electron microscope imaging (SEM). A chromatographic method (HPLC-DAD) was applied to assess changes in the contents of the analogs in the presence of their impurities. The study showed that the structures of the analogs did not demonstrate any significant alterations at the end of the period necessary for the elimination of unstable free radicals. The separation of main substances and related substances (impurities and potential degradation products) allowed determining that no statistically significant changes in the content of particular active substances occurred and that their conversion due to the presence of free radicals resulting from exposure to an irradiation of 25 kGy (prescribed to ensure sterility) was not observed.

## 1. Introduction

**Figure 1:**
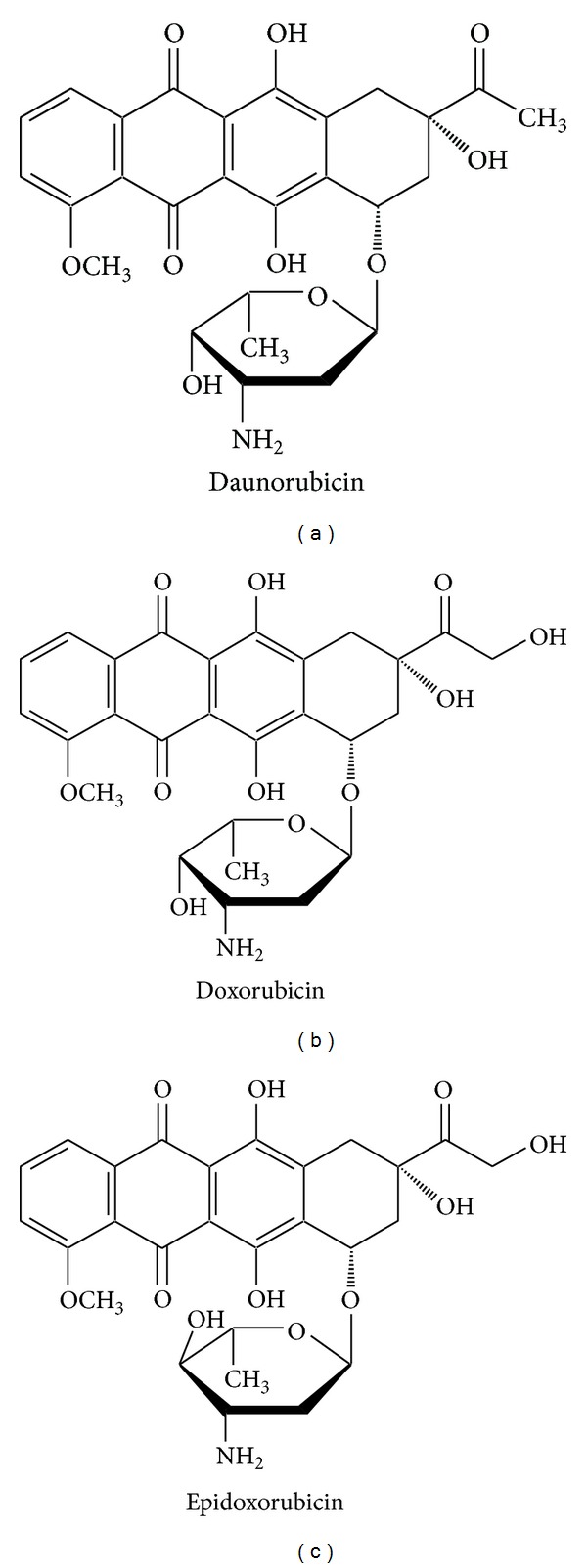
Chemical structures of daunorubicin, doxorubicin, and epidoxorubicin.

Anthracycline antibiotics belong to the group of anticancer drugs. They were originally isolated from cultures of *Streptomyces*. Anthracyclines are widely used in the treatment of neoplastic diseases such as leukemia, breast cancer, and AIDS-related Kaposi's sarcoma. They also demonstrate activity against tumors of the ovaries, lung, testes, prostate, cervix, bladder, and Ewing's sarcoma [1–3]. The most widely used anthracyclines are doxorubicin (DOX), daunorubicin (DAU), and epidoxorubicin (EPI) [4–6] (Figure 1). Their low bioavailability necessitates parenteral administration [7, 8], which requires sterility [9] obtained mainly by filtration. Given the considerable exposure of medical personnel to anthracycline antibiotics and their adsorption to most surfaces, especially those of sterilization filters, there is a need to find new sterilization methods that do not rely on filtration. Although radiation sterilization is an effective alternative, the structure of drugs may alter as a consequence of exposure to irradiation. The aglycone attached to the aminosugar with a glycoside bond may be prone to cleavage resulting from electron transfer within the molecule. Stability studies demonstrated degradation of anthracycline antibiotics in solutions, under the influence of increased temperature and when exposed to light in the solid state as well as a consequence of combining chemotherapy with radiotherapy [10–15]. Regarding the effects of radiation sterilization on anthracycline antibiotics, only DOX was studied in that respect with a focus on the impact of a standard dose of 25 kGy on its stability [16]. As DAU and EPI are widely used in anticancer pharmacotherapy, those antibiotics also require analysis of their vulnerability to ionizing radiation.

The aim of this work was to assess the possibility of applying radiation sterilization to DOX, DAU, and EPI as active substances or conversion-induced impurities.

## 2. Materials and Methods

### 2.1. Samples

DOX, DAU, and EPI were synthesized at the Institute of Biotechnology and Antibiotics, Department of Modified Antibiotics, Warsaw, Poland. They were reddish powders, freely soluble in water and methanol. Sodium lauryl sulfate, phosphoric acid, and all other chemicals were obtained from Merck KGaA (Germany) and were of analytical or high-performance liquid chromatographic grade.

### 2.2. Irradiation

0.025 g samples of each substance were placed in 3 mL colorless glass vials that were closed with plastic stoppers. The samples in the vials were exposed to beta irradiation in a linear electron accelerator LAE 13/9 (9.96 MeV electron beam and 6.2 *μ*A current intensity) until they absorbed doses of 25, 50, 100, 200, and 400 kGy.

### 2.3. Electron Paramagnetic Resonance (EPR) Spectroscopy

Detection of free radicals and determination of their concentration were carried out using a Bruker ELEXSYS 500 spectrometer (X-band) at 297 K. EPR spectra were recorded as a first derivative of the absorption signal. The number of free radicals was calculated using the integration procedure described elsewhere [17].

### 2.4. UV-Vis Spectroscopy

Chemical changes in nonirradiated and irradiated samples were analyzed by using a UV-Vis Varian Carry 100 spectrophotometer. 2 mg of each sample was dissolved in 20.0 mL of methanol. 1.0 mL of the so-obtained solution was diluted to 10.0 mL with methanol. The final concentration of the solutions was 0.01 mg/mL. Absorption spectra of the so-prepared solutions were recorded in the wavelength range 190–900 nm.

### 2.5. IR Spectroscopy

IR spectra of nonirradiated and irradiated anthracyclines were taken with the use of a Thermo Scientific Nicolet iS10 spectrophotometer with the Omnic software. The infrared transmittance spectra of the crystalline samples were recorded after a time necessary to achieve plateaus in EPR study, in the frequency range from 400 to 7500 cm^−1^, at room temperature.

### 2.6. HPLC Analysis

An HPLC Waters Alliance e2695 system was used for chromatographic separation of the degradation products of nonirradiated and irradiated DOX, DAU and EPI samples. All the samples (1 mg/mL) were dissolved in the mobile phase. A Symmetry C18 (250 × 4.6 mm, 5 *μ*m) analytical column was employed as a stationary phase. The mobile phase consisted of solution A (acetonitrile) and solution B (2.88 g of sodium lauryl sulfate and 2.25 g of phosphoric acid(*V*) 85% in 1000 mL) (50 : 50, v : v). UV detection was performed at 254 nm. The flow rate was 1.0 mL/min. The injected volume was 5 *μ*L.

### 2.7. Theoretical Analysis

All the calculations were made by using the Gaussian 03 package [18]. In order to interpret the experimental results of IR absorption scattering, quantum chemical calculations were performed based on a density functional theory (DFT) method with the B3LYP hybrid functional and 6-31G(d,p) basis set.

## 3. Results and Discussions

The first EPR analysis was performed 1 day after irradiation for samples exposed to a dose of 25 kGy. DOX, DAU, and EPI irradiated at 25 kGy contained about 3.94 × 10^15^ spins/g, 1.37 × 10^15^ spins/g, and 2.44 × 10^15^ spins/g, respectively. Exponential decay of unstable free radical was observed with the half-time of 4 days in DAU and DOX and 7 days in EPI after irradiation. The EPR signals of the sterilized anthracycline antibiotics were very weak. The plateaus of free radical concentrations versus time for DOX and DAU appeared after about 10 days, whereas for EPI after 20 days. EPR spectra after these periods consisted approximately of only stable free radicals.

**Figure 2:**
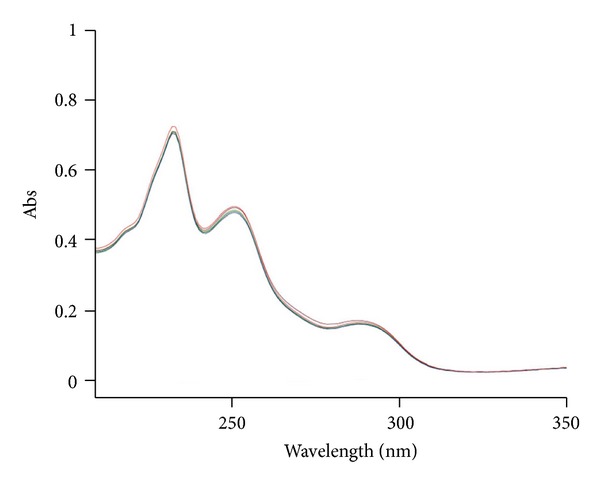
UV spectra of unirradiated and gamma irradiated daunorubicin.

**Figure 3:**
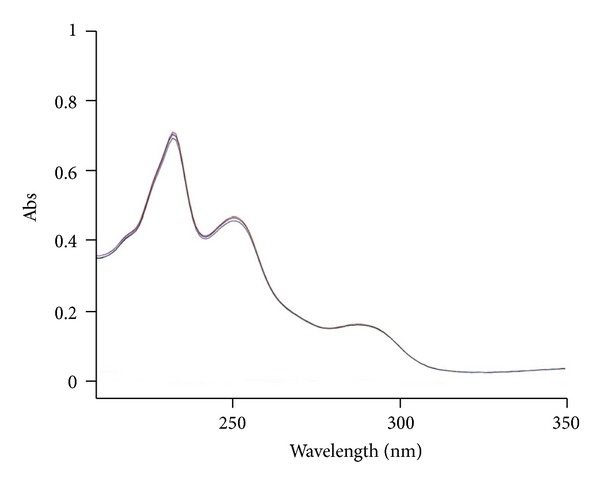
UV spectra of unirradiated and gamma irradiated doxorubicin.

**Figure 4:**
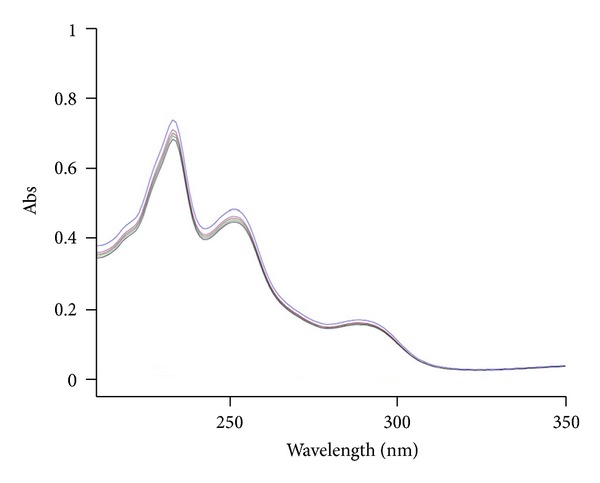
UV spectra of unirradiated and gamma irradiated epidoxorubicin.

**Figure 5:**
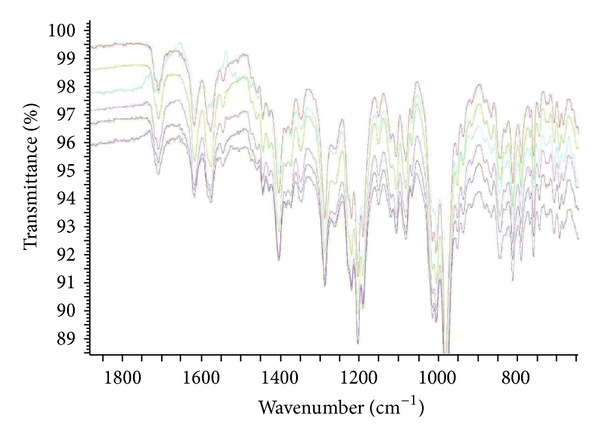
FT-IR spectra of unirradiated and gamma irradiated daunorubicin.

**Figure 6:**
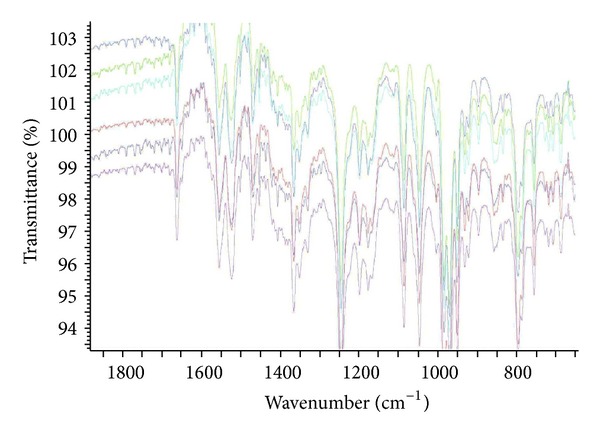
FT-IR spectra of unirradiated and gamma irradiated doxorubicin.

**Figure 7:**
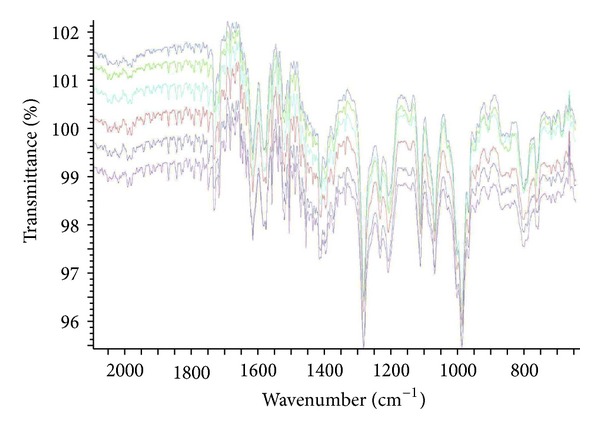
FT-IR spectra of unirradiated and gamma irradiated epidoxorubicin.

**Figure 8:**
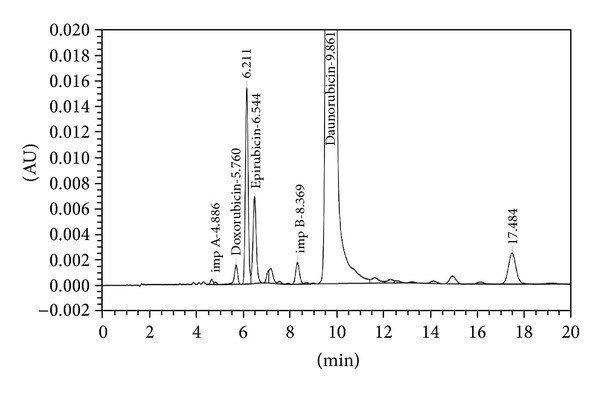
HPLC profile of unirradiated daunorubicin.

**Figure 9:**
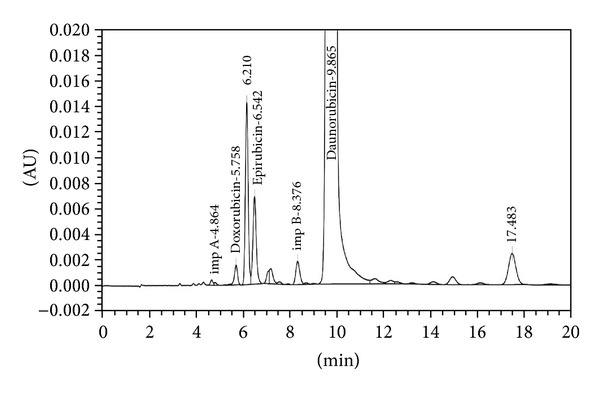
HPLC profile of irradiated daunorubicin (25 kGy).

**Figure 10:**
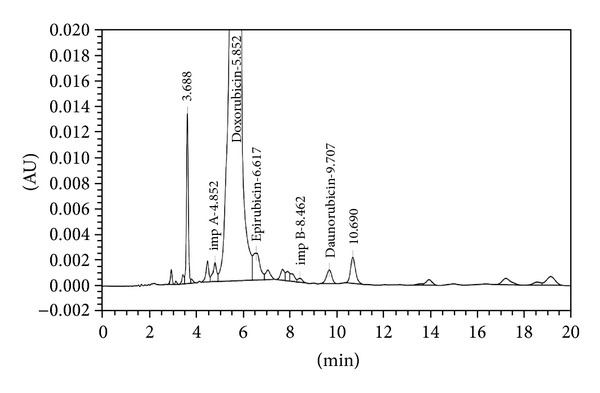
HPLC profile of unirradiated doxorubicin.

**Figure 11:**
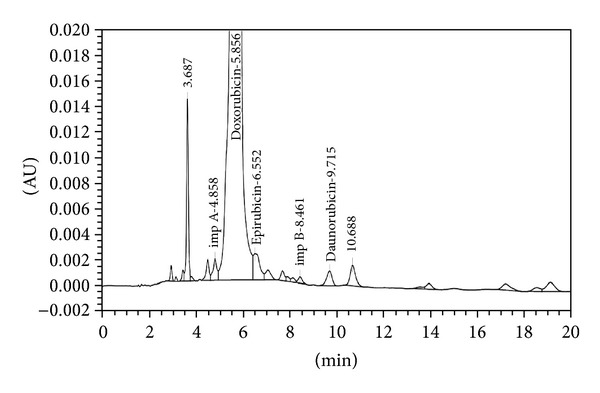
HPLC profile of irradiated doxorubicin (25 kGy).

**Figure 12:**
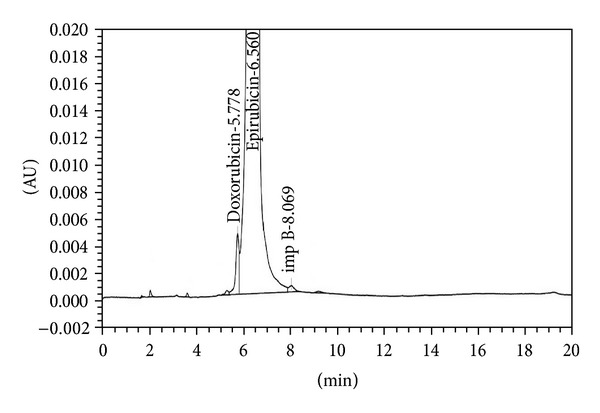
HPLC profile of unirradiated epidoxorubicin.

**Figure 13:**
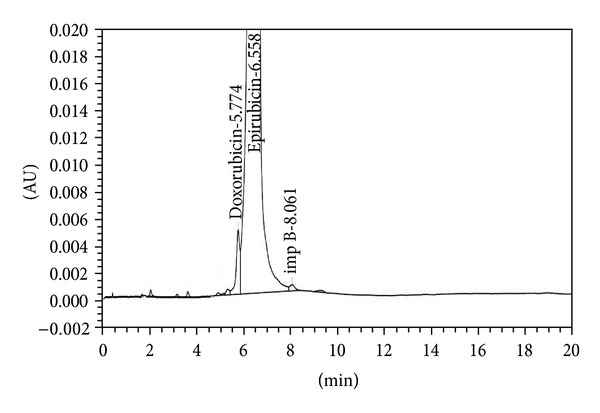
HPLC profile of irradiated epidoxorubicin (25 kGy).

The analytical study was conducted during a period when only stable free radicals were detected by EPR. The impact of an irradiation dose size on the structure of DAU, DOX, and EPI was studied at 0, 25, 50, 100, 200, and 400 kGy without the presence of unstable free radical in EPR spectra. By using UV-Vis spectroscopy the location and the intensity of the absorption maximum were determined, whereas IR spectroscopy was employed to establish the intensity, location, and type of characteristic vibrations. For the DAU, DOX, and EPI samples no significant changes in the location (~289 nm, ~233 nm, and 221 nm) or intensity of the absorption maximum were recorded (Figures 2, 3, and 4). The spectra of their nonirradiated and irradiated samples did not show any essential differences in the value of absorbance. All samples exhibited two absorption maxima, at 233 nm and 251 nm. The IR spectra of DOX, DAU, and EPI were compared with the theoretical spectra based on the density functional theory. The main characteristic vibrations obtained from the IR spectra are collected in Table 1. The conformation between the calculated and experimental spectra is quite good. The most significant is the region between 700 and 1800 cm^−1^, where intense and characteristic bands related to intramolecular vibrations of the molecules are observed, including the deformation of rings as well as stretching of various C–C bonds (Figures 5, 6, and 7). Vibrational spectra of the nonirradiated and irradiated samples of the three samples are very similar. We did not observe any change in the position and shape of the bands. This suggests that the radiation sterilization does not influence the stability of the DOX, DAU, and EPI. Similar results were received by comparing SEM images of the nonirradiated and irradiated samples. Taking into account the biological activity of the most important impurities specified by Ph. Eur. [9], changes in the concentration of the main substances in the presence of those impurities were analyzed (Figures 8, 9, 10, 11, 12, and 13). By separating the compounds to be examined from the impurities, it was possible to assess changes in their content before and after irradiation at 25 kGy. It was found that exposure to such a dose of radiation did not produce any changes in the concentrations of the main substances or the impurities.

Slight alterations were registered when the samples of DAU, DOX, and EPI were exposed to greater doses of radiation. Under such conditions, EPI demonstrated the greatest content change, and the presence of unstable free radicals was noted for the longest period of time. It was also proved, by observing the mass balance, that the main substances did not convert into unknown impurities. Similar studies of some tetracycline analogs showed that the aglycon was stable when irradiated at 25 kGy and that changes occurred when greater radiation doses were applied [19]. It may therefore be proposed that not only a modification of the aglycon structure but also its ability to bind with a sugar moiety of specific stereoisomerism are the factors that stabilize the structures of analogs of anthracycline antibiotics.

## 4. Conclusions

The current study of the impact of radiation sterilization on the stability of DAU, DOX, and EPI demonstrates that this kind of sterilization may be an alternative to filtration recommended for sterilizing analogs of anthracycline antibiotics. The effect of radiation sterilization on the stability of DAU, DOX, and EPI depends on the structure of a particular compound. With regard to those analogs, it is important to assay the postirradiation content of the main substance in the presence of all possible related substances in order to determine whether other degradation products or postirradiation conversion occur.

## Figures and Tables

**Table 1:** Main characteristic vibrational modes of daunorubicin (DAU), doxorubicin (DOX), and epidoxorubicin (EPI) observed in experimental and calculated spectra.

Calculation (cm^−1^)			Experimental (cm^−1^)			Band assignment
DAU	DOX	EPI	DAU	DOX	EPI	
723	736	736	764	761	761	C–C–C *b* in a ring + def. aglycone group + breathing tetrahydropyran ring in aminosugar group + NH_2_ *w*

752	754	749	795	795	795	NH_2_ *w* + C–C–C *b* in aminosugar group + C–H *w* at **d** ring and in aminosugar group

776	773	774	816	804	805	C–C–C *b* in a ring + def. aglycone group + breathing tetrahydropyran ring in aminosugar group + CH_2_ *r* at **d** ring

931	930	935	940	939	939	C–O–H *b* at c ring + C–H *w* in aminosugar group

959	957	951	955	949	949	C–C–C *b* in **d** ring + NH_2_ *w* + CH *w* at **d** ring and aminosugar group

984	—	—				CH_3_ *w* in COCH_3_ group

1010	1008	1006	986	990	989	Breathing aglycone group + CH_2_ *r* + NH_2_ *r* + CH_3_ *w* in COCH_3_ + CH_2_ *w* in COCH_2_OH

1021	1029	1032	1008	1005	1005	Breathing **a** ring + def. aglycone group + C–O *s* at **a** ring + C–O–H *b* at **c** ring + NH_2_ *w* + CH_3_ *w* in COCH_3_ group

1079	1078	1065	1070	1072	1072	C–O *s* between tetrahydropyran ring and O–H group in aminosugar group

1105	1104	1104	1085	1089	1089	C–O *s* in metoxy group at **a** ring + C–C–C *b* in **a** ring + CH_2_ *r *

1123	1123	1122	1109	1114	1114	C–O *s* in tetrahydropyran ring + C–O *s* in glycosidic bond + C–C *s* in **d** ring + C–H *w* in CH_3_ in aminosugar group

1137	1139	1136	1109	1114	1114	C–C *s* in **d** ring and in tetrahydropyran ring in aminosugar group + C–H *b* at **d** ring and in tetrahydropyran ring in aminosugar group + CH_3_ *w* in COCH_3_ group + C–O *s* in COCH_2_OH group

1157	1157	1156	1153	1143	1143	C–O *s* in glycosidic bond + C–O *s* between tetrahydropyran ring and O–H group in aminosugar group + C–H *r* in CH_3_ in aminosugar group + def. **d **ring

1206	1205	1200	1194	1201	1201	C–O *s* in glycosidic bond + C–C *s* I d ring + C–H *b* at **d** ring and aminosugar group

1226	1225	1221	1205	1211	1211	CH_2_ *t* at **d** ring + breathing **a** ring + C–O–H *b* at **c** ring

—	1239	1239		1235	1235	C–O–H *b* in COCH_2_OH group

1278	1275	1275	1262	1263	1263	C–O–H *b* at c ring + C–C *s* in aglycone group + CH_2_ *t* in COCH_2_OH group

1291	1290	1293	1289	1284	1285	C–O–H *b* at **c** ring + breathing **a** and **c** ring

1317	1317	1318	1289	1284	1285	Breathing **c** ring + C–O *s* at **c** ring + C–O *s* at **a** ring + CH_2_ *w* at **a** ring +C–H *w* at **d** ring

1329	1329	1330	1317	1318	1318	C–O *s* at a ring + breathing **a** and **c** ring

1367	1367	1366	1374	1374	1374	C–C–C *b* in **d** ring + C–C *s* in **a** and **b** ring + C–O *s* at **c** ring + C–O– *b* at **c** ring

1404	—	—				CH_3_ sc in COCH_3_ group

1425	1427	1423	1404	1413	1413	O–C–H *b* + N–C–H *b *

1476	1476	1478	1474	1471	1472	C–O *s* at c ring + def. **c** ring + CH_3_ *umbrella mode* at **a** ring + C–O–H *b* at **c** ring

1502	1502	1505	1506	1507	1507	C–O–H *b* at **c** ring + CH_3_ sc at **a** ring + C–C *s* in aglycone group

1524	1524	1527		1525	1524	C–O–H *b* at c ring + CH_3_ sc at **a** ring + C–C *s* in aglycone group

1614	1615	1615	1576	1582	1581	C–C *s* in **c** ring + C–O–H *b* at **c** ring + C=O *s* at **b** ring + C–C *s* in **a** ring

1640	1640	1642	1576	1582	1581	C–C *s* in **a** ring + C–O–H *b* at **c** ring + CH_3_ *w* at **a** ring

1661	1663	1663	1617	1616	1616	NH_2_ sc

1688	1688	1688	1617	1616	1616	C=O *s* at **b** ring + C–O–H *b* at **c** ring

1744	1744	1745	1707	1717	1717	C=O *s* at **b** ring

1791	1808	1807	1716	1730	1730	C=O *s* at **d** ring

2967–3237			2844–3108			C–H *s *

3009	3010	2960	2878	2896	2896	O–H *s* at **c** ring

3488	3488	3485				N–H *s* symmetric

3566	3566	3571				N–H *s* antisymmetric

3630	3634	3639	3161	3326	3329	O–H *s *

3791	3788	3785		3527	3527	O–H *s *

3809	3801	—				O–H *s *

—	3831	3831		3545	3545	O–H *s* in COCH_2_OH group

Vibrational modes—*s*: stretching, *b*: bending, *w*: wagging, *sc*: scissoring, *r*: rocking, and *t*: twisting.
